# Theoretical Rationale for Design of Tasks in a Virtual Reality-Based Exergame for Rehabilitation Purposes

**DOI:** 10.3389/fnagi.2021.734223

**Published:** 2021-11-02

**Authors:** Lars Peder Vatshelle Bovim, Lauritz Valved, Bendik Bleikli, Atle Birger Geitung, Harald Soleim, Bård Bogen

**Affiliations:** ^1^SimArena Rehabilitation Lab, Department of Health and Functioning, Western Norway University of Applied Sciences, Bergen, Norway; ^2^The Vitality Centre for Children and Youth, Haukeland University Hospital, Bergen, Norway; ^3^Department of Computer Science, Electrical Engineering and Mathematical Sciences, Western Norway University of Applied Sciences, Bergen, Norway

**Keywords:** exergaming, theoretical rationale, gait rehabilitation, motor, cognitive, virtual reality

## Abstract

Virtual reality games are playing a greater role in rehabilitation settings. Previously, commercial games have dominated, but increasingly, bespoke games for specific rehabilitation contexts are emerging. Choice and design of tasks for VR-games are still not always clear, however; some games are designed to motivate and engage players, not necessarily with the facilitation of specific movements as a goal. Other games are designed specifically for the facilitation of specific movements. A theoretical background for the choice of tasks seems warranted. As an example, we use a game that was designed in our lab: VR Walk. Here, the player walks on a treadmill while wearing a head-mounted display showing a custom-made virtual environment. Tasks include walking on a glass bridge across a drop, obstacle avoidance, narrowing path, walking in virtual footsteps, memory, and selection tasks, and throwing and catching objects. Each task is designed according to research and theory from movement science, exercise science, and cognitive science. In this article, we discuss how for example walking across a glass bridge gives perceptual challenges that may be suitable for certain medical conditions, such as hearing loss, when perceptual abilities are strained to compensate for the hearing loss. In another example, walking in virtual footsteps may be seen as a motor and biomechanical constraint, where the double support phase and base of support can be manipulated, making the task beneficial for falls prevention. In a third example, memory and selection tasks may challenge individuals that have cognitive impairments. We posit that these theoretical considerations may be helpful for the choice of tasks and for the design of virtual reality games.

## Introduction

Exergaming can broadly be defined as “A game that involves physical exercise and that integrates motion-tracking technology that enables interaction with the game and real-time feedback of user’s performance” (Perez-Marcos, 2018). Over the last decades, there has been an increasing interest in using exergames for rehabilitation purposes, and systematic reviews show that exergaming may be beneficial for a variety of outcomes in different clinical populations (Mat Rosly et al., 2017; Page et al., 2017; Pacheco et al., 2020). Often, the ability to engage and motivate is pointed to as the main benefit of exergames: Players are caught up in solving the tasks of the game, and “forget” that they are moving. As such, it can be said that metabolic effects are the goal of the game. There is also great potential in designing games and tasks that are directed towards specific impairments, both physical or cognitive (de Bruin et al., 2010; Vogt et al., 2019). The content and characteristics of the games, therefore, seem important for facilitating specific domains that can be beneficial in rehabilitation. However, the theoretical rationale for tasks chosen in exergames is not always clear and declared (Anders et al., 2020).

In this non-empirical article, we aim to describe the practical approach for designing and developing an exergame that is played while treadmill walking, and discuss the theoretical rationale for choice and design of specific tasks. The game is meant to challenge motor and/or cognitive skills and is intended for rehabilitation purposes.

## Background

With an aging population, the burden on health care resources is expected to increase substantially (WHO, 2021). New digital technologies that can engage and motivate for movement and activity are emerging, with great potential for health care and rehabilitation services (McCaskey et al., 2018). Exergaming is one such technology that has been found to benefit cognitive functioning (Stanmore et al., 2017), enhance prefrontal brain activity (Eggenberger et al., 2016; Schättin et al., 2016), and improve physical and mental health (Xu et al., 2020). Patients are often seeking, or are being referred to rehabilitation for movement problems, such as poor balance and gait impairments. To train movement skills, rehabilitation professionals use physical equipment (obstacles, objects to grasp, etc.) and physical space. Virtual realities are, at least to some extent, less constrained by physical realities: Paths and patterns on the floor to navigate can be made virtually, and “objects” can be data animated and grasped using hand controllers. Tasks can be tailored to specific impairments: Virtual footprints can serve as cues for patients with Parkinson’s disease and freezing gait (Gómez-Jordana et al., 2018), while the balance can be promoted using weight shifting tasks in persons with hemiparesis after stroke (Wüest et al., 2014). Fall prevention in older adults is an important issue in health care, and exercise is likely the most important all-round preventive measure that can be taken (Gillespie et al., 2012). Further, the most successful exercise programs to prevent falls are high-intensity balance exercises, meaning reducing the base of support, shifting of the center of mass, and little hand support (Sherrington et al., 2017). Mirelman and co-authors used a screen-based game that was played while walking on a treadmill in a randomized controlled trial of fall prevention and reduced the number of falls significantly compared to treadmill walking alone (Mirelman et al., 2016). The game involved stepping over and around obstacles (Mirelman et al., 2011), and thus involved narrowing the base of support and shifting of the center of mass. Importantly, the game involved both cognitive (navigation, planning) and motor processes (walking), which the authors argue are key to avoiding falls.

Exergames may be delivered through different platforms, such as desktop, CAVE, or head-mounted displays (HMD). One of the key differences between the platforms is the degree of immersiveness: While playing on a screen, the player is aware of her surroundings and the sensation of being in the game may be less pronounced than when wearing an HMD, which blocks out other visual (and auditory) stimuli. There are few studies comparing the platforms directly, but there is some research to suggest that memory performance within experimental tasks is enhanced in an immersive virtual environment compared to a non-immersive virtual environment (Ventura et al., 2019). More immersive platforms, therefore, show greater promise as a means of rehabilitation.

At our lab, the exergame VR Walk was developed in close collaboration between computer scientists and physiotherapists. The aim of the game is to improve balance and mobility for different clinical groups through engaging and motivating motor-cognitive training. As a guiding principle, we used training conditions based on Geurts and co-authors (Geurts et al., 1991), by adding difficulties to the basic task of walking:

Perceptual: Altering perceptual input (in this game, only visual).

Cognitive: Performing more than one task at the same time.

Motor: Increasing the dynamic complexity of a basic task, such as turning and throwing.

Mechanical: Changing and decreasing the base of support.

An early version of VR Walk (the minimal viable product) has been tried on healthy adults, where we investigated how the different tasks changed gait regularity (Bovim et al., 2020), and the game is planned for use in projects with both children/adolescents and older adults.

## Materials and Methods

An iterative design approach is defined and used in this project, within an interprofession team including health (scientists and clinicians) and computer science (Still and Crane, 2016). Weekly user tests were arranged during planning and development phases, while validation and evaluation were performed over a longer time frame. The project was undertaken between 2018–2020. In overview, the project process can be presented in the following five phases;

1. Design and defining
The initial aim was to design an HMD-based exergame that would challenge balance, and involve both motor and cognitive skills simultaneously. The idea for the game came from the first author (LPVB), who then pitched the project to the Department of Computer Science. Two students (LV&BBI) and two supervisors (HS&AG) from computer science were included in the project. From the outset, the aim was to make a game that could be played when walking on a treadmill, and this was a guiding principle for the rest of the design.
2. Prototyping
A minimal viable product (MVP) was produced, following an iterative approach, including weekly proto-testing and adjusting and stage-based ease of use-testing. The MVP included a base of tasks, focusing on perceptual-, motor-, and mechanical constraints (Bovim et al., 2020). The MVP was then tested by investigating how gait parameters changed when executing the tasks of the game in a sample of healthy adults.
3. Validating
The MVP was tested on 29 healthy, young adults (mean age 29 years old). Participants wore an accelerometer on their lower back to capture gait parameters (step length, cadence, walk ratio, and stride regularity in the anteroposterior, mediolateral, and vertical directions). As anticipated, gait patterns changed according to task: For example, regularity was low during the coin-catching task as the center of mass was shifted randomly to reach the coins, and regularity was high when participants used a more rigid movement pattern when balancing the ball. There was no increase in simulator sickness (Wüest et al., 2014). The validation phase was executed as a master thesis of Physiotherapy.
4. Evaluation
Using the MVP as inspiration, an end-user panel of clinicians from the fields of physiotherapy, neurological rehabilitation, and psychiatry were included in a workshop focusing on potential tasks aiming towards their field of expertise as a part of a master thesis of Computer Sciences.
5. Design and development phase
Based on the experiences from validation and evaluation, a new version of the game was developed, using a similar iterative approach as during prototyping, with some new tasks aimed at training motor-cognitive skills.

## Results

**Figure 1:**
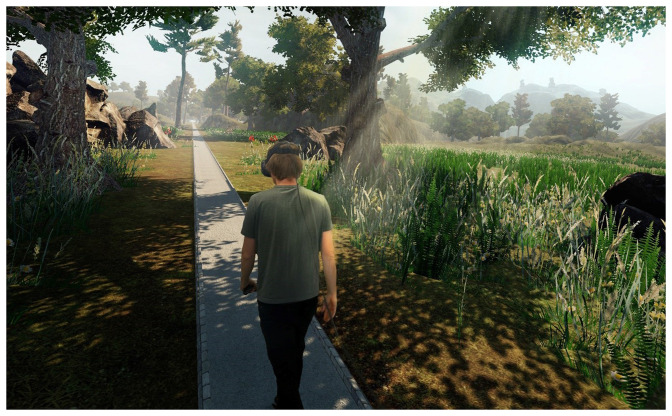
The virtual environment of VR Walk is set to a Mediterranian terrain.

In 2020, the present version of VR Walk was finished. This is an HMD-based VR game designed for use on a treadmill. The game is set in an environment that resembles a Mediterranean area (Figure 1), and the main objective is to walk on a virtual path while avoiding obstacles and performing tasks of perceptual, cognitive, and motor characteristics (See “Tasks” section). A dynamic avatar of hand(s) and feet are included using hand controllers and HTC Tracker 2.0, replicating the user’s real time movements of hands and feet. For all tasks except “Footsteps”, only one hand controller is needed. Treadmill speed can be adjusted by either the player or therapist, with adjustment rates of 0.1 km/h. The game is only available using tethered VR, such as HTC Vive, Oculus Rift, etc.

### Safety Measures

**Figure 2:**
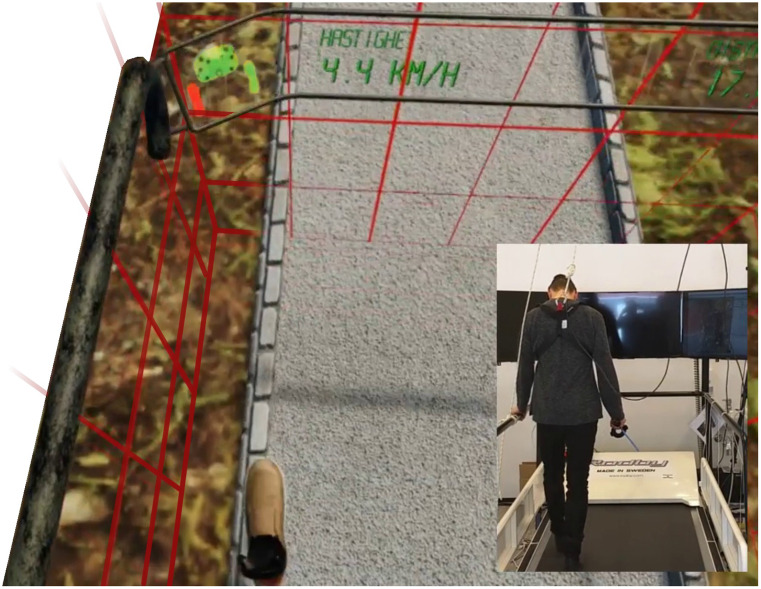
When the player steps toward the limits of the game area, a grid is visualized. If the player steps past the game area, the grid turns red and the treadmill stops automatically. External safety measures such as harness and treadmill railing is recommended.

Several safety measures are embedded in the game. For the novel player, a virtual representation of the treadmill handrails is always visible for the player to grab on to when necessary. An experienced player may hide these for increased immersion. If the player is disoriented and heading towards one of the edges of the treadmill, a virtual grid-pattern appears, indicating the player to adjust the position. Treadmill handrails will also re-appear. The grid-pattern will disappear if the positioning is adjusted. If the player’s position falls outside the defined area of the grid-pattern, the treadmill will automatically stop, and the player is requested to return to the “safe area” before the treadmill can be started again (Figure 2).

In addition to software features, external safety measures such as using a non-weight-bearing harness and close supervision are highly recommended, and each player is given thorough instructions (how to operate the hand controller(s), visual inspection of safety measures, etc.). New players also walk for a certain distance without any additional tasks, for familiarization with the virtual environment and treadmill walking.

### Tasks

**Figure 3:**
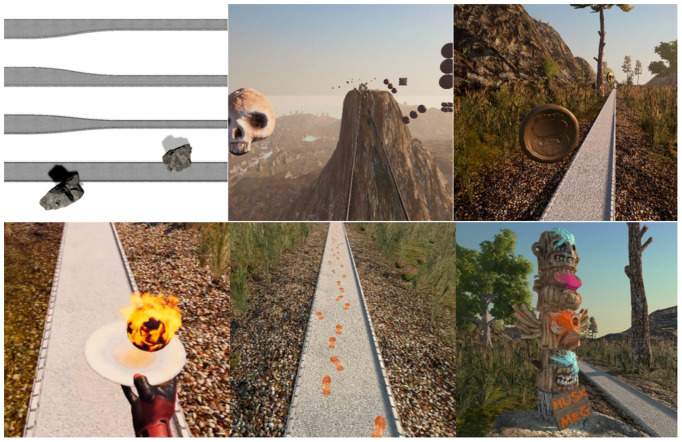
The tasks of VR Walk (from upper left): Path adjustments. Glass bridge (with axe throwing). Coin collection. Balance ball. Footsteps. Memorize.

Based on the iterative design approach, six tasks with difficulty adjustments were developed (Figure 3);

1. *Full path*: The width of the path is equal to the treadmill width (100 cm).
Increased difficulty: The width of the path is reduced by 30, 50, and 70 percent, or obstacles of large rocks can be placed on the path.
2. *Glass bridge*: A semi-transparent glass bridge simulated to appear approx. 200 m above the ground.
Increased difficulty: Axe throwing at clusters of spheres that hang in the air, and “selective balls”, where spheres that are shaped like skulls should be avoided, can be selected by the therapist.
3. *Coin collecting*: The player approaches coins that swirl in the air. The task is to catch the coins using the hand controller.
Increased difficulty: Movement pattern and speed of coins can be adjusted from stillstand to extreme movement (2 m sideway and 1.5 m up/down), and “selective coins”, where only gold coins are to be collected, can be selected by the therapist.
4. *Balance ball*: The player is holding a virtual disc in the hand with the hand controller. A virtual ball appears on the disc, and the player must balance the ball on the disc.
Increased difficulty: The ball is on fire, and will melt the disc unless it is being bounced off the disc.
5. *Footsteps*: Virtual footsteps appear on the path, and the player is instructed to step on the footsteps. This task requires the player to wear foot trackers.
Increased difficulty: The step length, step width, and between-step variability can be increased and decreased.
6. *Memorize*: A totem pole stationed by the path is to be memorized, and later physically puzzled in the correct order.
Increased difficulty: Hight can be adjusted from 1–4 pieces, and the pieces may be individually painted in separate colors.

The player is given positive visual, audible, or/and haptic feedback on correctly performed tasks, and negative visual, audible, and haptic feedback when tasks are performed incorrectly. At the end of the game, a total score and subscores of each task are given.

### User Interface

The main menu of VR Walk, with basic steps such as “start”, “options” and “credits”, is accessible both for the therapist *via* desktop and for the player directly in the HMDs. The desktop menu makes use of Unity’s UI toolkit. Among other things, the toolkit includes buttons, sliders, toggles, and text fields, all of which can execute, and be accessed by, custom scripts. The therapist can use a standard mouse and keyboard. The menu accessible from the HMDs is placed in a world space position, and a laser pointer from the SteamVR plug-in is used to allow the player to interact with the UI elements.

In-game, the player can access a treadmill panel using said laser pointer. The treadmill panel is added to the visual representation of the treadmill’s handrails and includes basic adjustments of speed and start/stop, live statistics of current speed, distance walked and distance remaining, and score of on-going tasks.

The desktop-based “therapist menu” includes the functions of “calibration” and “path builder”.

### Calibration

**Figure 4:**
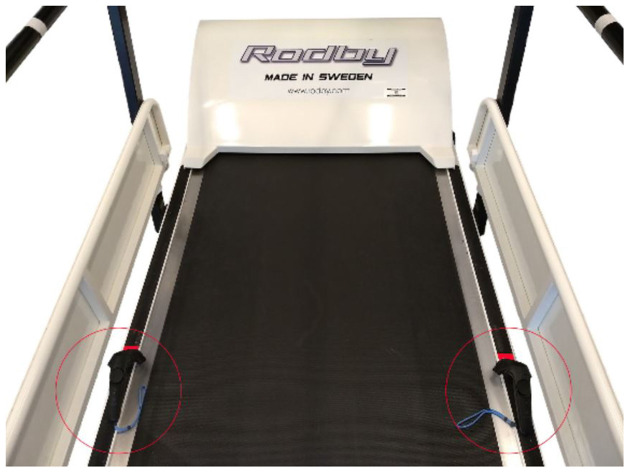
During calibration process, the hand controllers are physically placed on the treadmill as illustrated.

The calibration process takes full advantage of the precise positional data of the Lighthouse-tracked motion controllers. The controllers are placed on symmetrical markers on either side of the treadmill belt and the controller positions are registered (Figure 4). A vector is drawn between the two registered points. The cross product between this vector and a vector facing directly upwards in the virtual environment yields a new vector that is parallel to the treadmill band. This resulting vector is then reversed or left unchanged depending on the orientation and left/right assignment of the controllers.

### Path Builder

**Figure 5:**
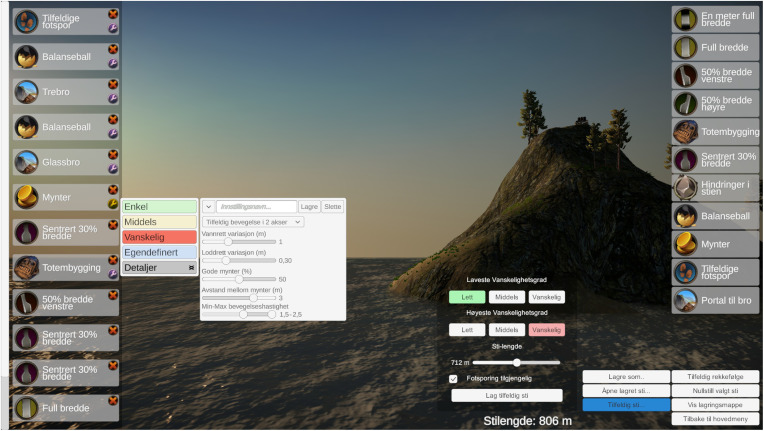
The therapist/operator can access a detailed Path Builder and design individualized tasks and paths.

The therapist can individualize paths using the Path builder (Figure 5). Here, all tasks can be added in chosen or random order. Further, each task can be defined using pre-defined settings of easy, medium, and hard, or in detail using added sliders. Detailed defined tasks can be saved for re-use later, as can full paths.

## Discussion

We have described an HMD-delivered exergame designed for treadmill walking. In this section, we discuss the elements of the game against relevant literature. First, we discuss some of the overall aspects of VR Walk, and then we discuss the specific tasks of the game.

VR Walk is situated in a life-like environment, with plants moving slightly in the breeze and mountains in the background, with realistic textures on the features of the game. The aim was to provide an attractive and soothing backdrop for the game and to add to the presence of the experience. Other games use more stylistic backgrounds, where the elements of the game are at the forefront (Giphart et al., 2007; Skjæret et al., 2015). It can be argued that realistic surroundings can be distracting, and that “cleaner” designs allow the player to focus on the tasks. It could also be argued that realistic environments would allow for greater transferability to real-life situations. This is in line with principle no. 3 of Kleim and Jones’ principles of neuroplasticity (Kleim and Jones, 2008), that the nature of the training experience dictates the nature of the plasticity. However, this is debatable regarding VR Walk, as it takes place in an environment that is specific to very few people. Busy streetscapes or suburban landscapes would likely be more relevant to the majority of players and should be considered in further updates.

When playing VR Walk, the player must walk. This is not entirely novel in exergaming contexts. Mirelman et al. (2016) and Shema-Shiratzky et al. (2019) have used exergaming during walking for both fall prevention purposes in older adults, and for cognitive and social functioning in adolescents with ADHD. The novelty of VR Walk is that the virtual environment is delivered through HMDs, making for a more immersive experience, and to our knowledge, very few games using natural walking (overground or treadmill) and HMDs exist. Although walking is often regarded as a relatively automated task, attention is still required, as evidenced by detriments in walking performance while doing an additional task simultaneously (Paul et al., 2005; Clark, 2015). Performing dual tasks, such as walking and exergaming can therefore be called “motor-cognitive training” (Mirelman et al., 2016; Herold et al., 2018). VR walk is a game that is designed for being played when walking, and as such should lend itself to gait rehabilitation purposes. However, it could also be argued that the game implicitly is a dual-task exercise, as all the tasks in the game are added to the task of walking. By this reasoning, the walking part can be seen as a tool for practicing complex tasks, which can be beneficial for many contexts, not just those where walking is involved.

On a general note, all the tasks of the game require anticipatory balance control, meaning that the players can adapt proactively and continuously to the tasks of the game. None of the tasks challenge reactive balance control, where the player would have to regain balance after unexpected perturbations (Winter, 1995). Reactive balance control has been found to be associated with falls in older stroke survivors (Mansfield et al., 2015). Virtual environment-based games offer the possibility for reactive balance control tasks in a safe environment: Objects could appear from different directions at random intervals, forcing the player to make quick avoidance movements. For further development of VR Walk, this option might be explored.

On a similar note, uncertainty is not a factor that has been explored in this exergame. Uncertainty is a much-used feature to increase engagement in games and can refer to for example unpredictability (uncertainty about what will happen next in the game element) or randomness (uncertainty about the order of what will happen in the game element; Li et al., 2020). Xu and co-authors showed that adding uncertainty in the game elements increased physical exertion in players (ibid). It could be argued that some of the tasks of VR Walk contain uncertainty, such as the flying-coins task, but uncertainty is not a feature that is systematically embedded in the game. Future versions of the game could use tasks that used for example unpredictability and randomness to a greater extent, and we are currently working on adding human figures that appear from the side or suddenly rise up to the environment. It should also be noted that the game is intended for clinical groups and older adults that may not have extensive gaming experience generally, and virtual reality experience specifically, and it may not be necessary to add many elements that increase exertion.

In the MVP of VR Walk, points were not awarded for the completion of the tasks, and there was very little possibility for progression of difficulty. We have not found studies that show whether player-engagement is enhanced by scoring and progression in rehabilitation, but intuitively, scoring systems and increasing difficulty are important for not getting bored. Studies of gamers show that certain features are important for video games to become engaging, such as challenges and appropriate feedback (Altamimi and Skinner, 2012). Although the context of rehabilitation is different than that of gaming for entertainment, at the core is still the ability of the game to engage and motivate users. We, therefore, view scoring, haptic feedback, and progression of difficulty as an important aspect of the game, both from a motivational point of view and for tracking of progression. Also, progression of difficulty is vital for individualizing the tasks.

### The Tasks and Their Rationale

In this section, we will discuss the specific tasks.

Players who are naive to the game or to immersive VR start with a familiarization walk without constraints or tasks. In data presented as conference proceedings, we found immediately increased gait variability when walking on a treadmill with HMD compared to walking without in adults. However, gait patterns returned to normal in a relatively short time, showing that healthy adults adapted well to walking with HMDs (Bovim et al., 2019).

#### Path Adjustment

In the first task, the path narrows down by 30, 50, and 70 percent. In mechanistic terms, balance is defined as keeping the line of gravity within the base of support (Pollock et al., 2000). During walking, the line of gravity routinely falls outside of the base of support when one foot is off the ground (Mansfield et al., 2015). By making the path narrower, the ability of wider step width is reduced, potentially making the base of support smaller also during double support. We have not found studies where the effect of narrow walking is studied alone or specifically with regards to reduction of fall risk or to improve balance. However, manipulation of the base of support is common in exercise programs focusing on for example fall prevention. In a systematic review, Sherrington et al. (2017) report that for fall prevention exercises to be successful, they need to be intensive and challenging. One of the features of challenging exercises was reported to be a small base of support. It could be argued that at 30 percent, the path is still 30 cm wide and that older adults tend to walk with step widths of approximately 10 cm or less (Hollman et al., 2011). Thus, there is potential for making this task even more challenging.

In experimental conditions, older adults perform worse at obstacle avoidance tasks than young adults (Galna et al., 2009; Chen et al., 2017), suggesting that this is a task that is relevant for rehabilitation settings (e.g., increasing balance or cognitive capacity). However, the nature of treadmill walking is to walk in one direction. We have included a condition where the player is to pass large rocks in the path, by stepping to the side, but it is our experience that players who have tried the game do not find this very difficult. It should be noted that Mirelman et al. (2016) used an obstacle negotiation task that involved stepping over virtual objects, which proved efficacious in preventing falls in older adults, and this is easier to integrate into a straight walking paradigm.

#### Glass Bridge

Crossing the glass bridge is different from the other tasks, as no other effort than walking is required. However, height exposure in virtual reality can be perceived as stressful as in physical reality (Simeonov et al., 2005), so walking can be seen as a challenge in itself. We have not found any studies on fall or balance outcomes after height exposure in virtual reality, but Peterson et al. (2018) have found that walking on a beam with virtual height exposure decreased dynamic balance and increased cognitive load in young adults, suggesting that this condition was challenging and could have a training effect. We found that gait regularity and walk ratio decreased when participants walked onto the glass bridge, i.e., they walked more unsteady and cautiously, but also that these gait characteristics returned to normal before walking off the bridge, suggesting that people adapt quickly (Bovim et al., 2020). For progression, players can throw an ax at objects that hang in the air. This necessitates players to look away from the drop and focus on the spheres, and as such, the height exposure feels less precarious and is really a different task more than a progression of difficulty. A better progression of the height exposure challenge would possibly be to start at lower heights and then gradually increase the height.

#### Coin Collecting

The coin-collecting task requires the player to touch flying and swirling coins with the hand-controller. This task is meant as a motor task, placing demands on eye-hand coordination (and is an added task to the task of walking). In the validating phase, the coin-catching task was the task that induced the most gait variability on the participant (Bovim et al., 2020). We suggest that due to the nature of the task, where coins come at different heights and at both sides of the player, and the player has to reach for the coins, the center of gravity is being continually shifted in the direction of the reaching arm, leading to larger stride-to-stride fluctuations than the other tasks. As such, the task has considerable biomechanical aspects. At high levels of difficulty, the coins follow largely unpredictable paths, adding some similarity to reactive balance control training. Further, it is possible to add a response-inhibition-task, where only golden coins should be touched, and gray skulls should be avoided. This is an additional effort that puts demand on executive function. Executive functioning is higher-order mental processing that allows us to concentrate, to self-regulate, and to adapt to new circumstances (Theill et al., 2011). Reduced executive functioning is associated with gait dysfunction and falls in older adults (Zhang et al., 2019), and is an important target for fall prevention strategies (Liu-Ambrose et al., 2013).

#### Balance Ball

The balance ball task is a manual task that involves keeping a ball on a disc that is held in the hand by the hand controller. Like the coin collecting task, this task imposes the need for cognitive resources and eye-hand coordination. However, while the coin-collection task requires the player to reach in different, random directions, the balance ball task requires the player to adopt a rigid and cautious gait pattern, as seen in the validation phase where gait regularity in the anteroposterior direction increased substantially from the coin-collection task (Bovim et al., 2020). We have not found earlier research about balance- or mobility-related benefits from training by walking with a rigid gait pattern, but suggest that practicing different gaits for different tasks and contexts is beneficial for an overall ability to adjust physical behaviors to different situations.

#### Footsteps

In the footsteps-task, virtual footprints appear on the ground in front of the player, and the player is rewarded points for hitting the footsteps with his own feet. Progression of difficulty can be achieved by altering step width, step length and by making the sequence of footsteps more variable. Foot placement accuracy is lower in older adults and is a relevant task for example fall prevention exercises (Hoogkamer et al., 2017). Gómez-Jordana and co-authors (Gómez-Jordana et al., 2018) used a similar approach for patients with Parkinson’s disease, with the aim of improving gait performance by using the footprints as cues. In VR Walk, the difficulty is achieved by making gait patterns more challenging, in reality imposing gait variability upon the player. This task forces the player to make rapid shifts of the center of mass, and there is a component of reactive balance control to this task. It should be noted that there are some issues with the accuracy of the HTC foot trackers, for example such as large offsets when tracking is lost (Niehorster et al., 2017). This could mean that the physical hits with the feet do not match the virtual footprints entirely. This emphasizes the importance of thorough calibration of the game space and sufficient tracking (base stations and lighting).

#### Memorize

VR Walk includes a memory task; when walking along the path, a sign appears saying “Remember me”. The player then sees a totem pole with distinct heads on top of one another. Each head has a color. The heads then disappear, and after a 60 m walk (potentially including a separate task), the heads reappear, and the player has to stack them in the correct order, and give them a stroke of paint with a virtual paint brush with the correct color. The aim of this task is to challenge attentional capacity: The ability to walk and retain information in working memory at the same time. Dual task-exercising has been investigated in several studies, showing benefits on balance outcomes in multiple populations such as older adults and persons with neurological disorders (Fritz et al., 2015; Ghai et al., 2017; Martino Cinnera et al., 2021). The benefits are believed to arise from increased automatization of the motor task, and less need for executive resources (Clark, 2015; Fritz et al., 2015). The nature of the added task is important, and both semantic, arithmetic and manual tasks have been used in studies (Diamond, 2013; Leone et al., 2020). Nordin et al. (2010) showed that the cognitive-motor inference or dual task-cost of different added tasks (manual task, counting task, verbal task) had different impacts on gait parameters. In VR Walk, the additional task is to memorize two four shapes and three colors, and therefore relies more on processing and retaining visual information than the tasks in the aforementioned studies. Virtual environments are less bound by physical possibilities and allow for more diverse additional tasks than normal clinical settings, which in our opinion should be explored and refined further: For example, regarding this specific task in VR Walk, stacking and operating a paint brush add a manual aspect to the task. This could be considered a third task, which makes it quite complex. There is an argument that too high motor-cognitive interference may have negative effects in individuals with for example reduced cognitive capacity (Peterson et al., 2018). The inclusion of colors can therefore be removed for difficulty adjustment. Even less complex additional tasks could still be useful in later updates. In addition, the players are not given explicit instructions for prioritization, but the treadmill may give implicit prioritization; unless the player prioritizes walking, she will fall.

### Limitations

In this article, we describe and discuss the rationale for the design and choice of tasks for VR Walk, without any data to substantiate the discussion. The MVP of the exergame has been tried on a convenience sample of healthy adults, with the aim of investigating how the tasks would affect gait. However, it is not clear how the exergame would be accepted in a clinical population, nor whether it would be efficacious in improving motor- or cognitive skills. Also, although the difficulty of the game can be adjusted, we do not have firm knowledge about how increased difficulty affects the player. For example, does a 30, 50, or 70 percent narrower path make users 30, 50, or 70 percent more unstable? Based on our observations of people who have tried the game, instability during this particular task appears to change in a non-linear way; a path that is 30 and 50 percent narrower seems well accepted, while 70 percent appears to induce changes in locomotion (less regular walking). This could suggest that 30 and 50 percent represent an attractor state and that 70 percent represent a (literally unstable) phase transition (Van Hooren et al., 2018). However, as we have no real-life data, this is conjecture. It also emphasizes that careful consideration should be put into how difficulty is progressed or regressed in the game, especially as the aim of the game is to provide tasks that can be specific to specific conditions.

Also, the overall design process is not grounded in a specific model, it is only inspired by the wide concept of the iterative design approach.

The target group for the game are individuals who need training or exercise for health purposes. While exergaming has been found well accepted among for example older adults (Nawaz et al., 2016), there may still be barriers for rehabilitation providers in providing technological services. Glegg et al. (2013) investigated the adoption of virtual reality for rehabilitation after traumatic brain injury among rehabilitation therapists, and found that the therapists were positive and had intentions to use the method, but scored lower on self-efficacy. In a scoping review, Glegg and Levac (2018) show that lack of knowledge and skills, and beliefs about capabilities and consequences were barriers to using virtual reality games in rehabilitation. This emphasizes an important clinical aspect of the game: That regardless of how well it may perform, the downstream uptake among rehabilitation professionals may be low. Clinicians may be hesitant due to the perceived lack of technical skills. Also, using the game has a practical and financial side; a treadmill is required, in addition to an adequate computer and a safety harness, as well as a room where the equipment will fit. Although not outlined in this article, the developers are focusing on how to implement the game into clinical practice, such as giving workshops and courses and identifying super-users who are motivated and preferably have experiences with exergaming. Hopefully, this can be helpful for more extended use of the game.

## Conclusion and Clinical Implementation

In this article, we have described the development of the exergame VR Walk, and discuss theoretical considerations behind the tasks in the game. On a general note, we believe that the game can offer engaging and motivating motor-cognitive training for balance and mobility, increasing fellow researchers’ and clinicians’ possibilities to reproduce and implement future relevant findings. Neither the effectiveness nor the efficacy of VR Walk has been tested in trials yet, but we intend to use the game in a feasibility study of balance exercises in older adults with hearing impairment^1^, and we also plan to use it in a trial of children and adolescents with chronic disease. Further developments of the game are ongoing, using the design process that has been described.

## Data Availability Statement

The original contributions presented in the study are included in the article, further inquiries can be directed to the corresponding author.

## Author Contributions

All authors contributed relatively equal to the development and execution of the project. LPVB and BBo contributed with theraputic knowledge and relevance, and manuscript. LV and BBl contributed mainly on the development on MVP and VR Walk, with HS and AG as supervisors and key persons of knowledge within the field of computer sciences. All authors contributed to the article and approved the submitted version.

## Conflict of Interest

The authors declare that the research was conducted in the absence of any commercial or financial relationships that could be construed as a potential conflict of interest.

## Publisher’s Note

All claims expressed in this article are solely those of the authors and do not necessarily represent those of their affiliated organizations, or those of the publisher, the editors and the reviewers. Any product that may be evaluated in this article, or claim that may be made by its manufacturer, is not guaranteed or endorsed by the publisher.

## Footnotes

^1^ https://clinicaltrials.gov/ct2/show/NCT04283279
